# A monoclinic polymorph of dichlorido(2,4,6-tri-2-pyridyl-1,3,5-triazine-κ^3^ *N* ^2^,*N* ^1^,*N* ^6^)manganese(II)

**DOI:** 10.1107/S1600536811034118

**Published:** 2011-08-27

**Authors:** Kwang Ha

**Affiliations:** a School of Applied Chemical Engineering, The Research Institute of Catalysis, Chonnam National University, Gwangju 500-757, Republic of Korea

## Abstract

The Mn^II^ ion in the title complex, [MnCl_2_(C_18_H_12_N_6_)], is five-coordinated in a distorted square-pyramidal geometry by three N atoms of the tridentate 2,4,6-tri-2-pyridyl-1,3,5-triazine ligand and two chloride anions. In the crystal, the pyridyl rings are located approximately parallel to their carrier triazine ring, making dihedral angles of 5.0 (1), 3.8 (1) and 3.2 (1)°. Intramolecular C—H⋯N hydrogen bonds are present. The complexes are stacked in columns along the *c* axis and linked by inter­molecular C—H⋯Cl hydrogen bonds, forming one-dimensional chains. In the column, inter­molecular π–π inter­actions between the six-membered rings are present, the shortest centroid–centroid distance being 3.623 (2) Å. The structure reported herein represents a monoclinic polymorph of the previously reported triclinic form [Ha (2010 ▶). *Acta Cryst.* E**66**, m262].

## Related literature

For the triclinic crystal structure of the title complex, see: Ha (2010 ▶).
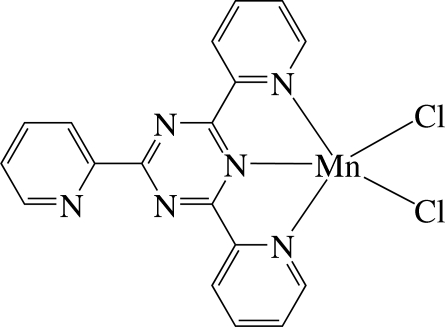


## Experimental

### 

#### Crystal data

[MnCl_2_(C_18_H_12_N_6_)]
*M* *_r_* = 438.18
Monoclinic, 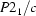

*a* = 10.8799 (9) Å
*b* = 19.5524 (16) Å
*c* = 8.6446 (7) Å
β = 101.051 (2)°
*V* = 1804.8 (3) Å^3^
*Z* = 4
Mo *K*α radiation
μ = 1.04 mm^−1^
*T* = 200 K
0.29 × 0.15 × 0.15 mm

#### Data collection

Bruker SMART 1000 CCD diffractometer
Absorption correction: multi-scan (*SADABS*; Bruker, 2000 ▶) *T*_min_ = 0.826, *T*_max_ = 1.000
13299 measured reflections
4450 independent reflections
2653 reflections with *I* > 2σ(*I*)
*R*_int_ = 0.056

#### Refinement

*R*[*F* ^2^ > 2σ(*F* ^2^)] = 0.045
*wR*(*F* ^2^) = 0.116
*S* = 1.01
4450 reflections
292 parameters
All H-atom parameters refined
Δρ_max_ = 0.52 e Å^−3^
Δρ_min_ = −0.56 e Å^−3^

### 

Data collection: *SMART* (Bruker, 2000 ▶); cell refinement: *SAINT* (Bruker, 2000 ▶); data reduction: *SAINT*; program(s) used to solve structure: *SHELXS97* (Sheldrick, 2008 ▶); program(s) used to refine structure: *SHELXL97* (Sheldrick, 2008 ▶); molecular graphics: *ORTEP-3* (Farrugia, 1997 ▶) and *PLATON* (Spek, 2009 ▶); software used to prepare material for publication: *SHELXL97*.

## Supplementary Material

Crystal structure: contains datablock(s) global. DOI: 10.1107/S1600536811034118/hp2014sup1.cif

Additional supplementary materials:  crystallographic information; 3D view; checkCIF report

## Figures and Tables

**Table 1:** Selected geometric parameters (Å, °)

Mn1—N1	2.189 (2)
Mn1—N4	2.318 (2)
Mn1—N6	2.348 (2)
Mn1—Cl1	2.3470 (10)
Mn1—Cl2	2.3400 (10)

**Table 2:** Hydrogen-bond geometry (Å, °)

*D*—H⋯*A*	*D*—H	H⋯*A*	*D*⋯*A*	*D*—H⋯*A*
C10—H10⋯Cl1^i^	0.91 (3)	2.76 (3)	3.589 (3)	151 (3)
C15—H15⋯Cl1^ii^	0.92 (3)	2.81 (3)	3.633 (4)	151 (2)
C3—H3⋯N2	0.97 (3)	2.52 (3)	2.896 (4)	103 (2)
C9—H9⋯N3	0.92 (3)	2.41 (3)	2.735 (4)	101 (2)
C15—H15⋯N3	0.92 (3)	2.57 (3)	2.869 (4)	100 (2)

Symmetry codes: (i) 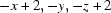; (ii) 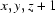.

**Fig. 1.**
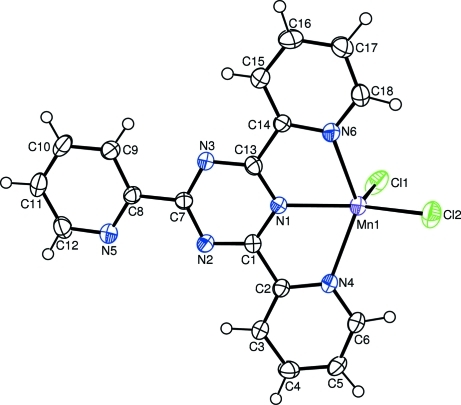
The structure of the title complex, with displacement ellipsoids drawn at the 50% probability level for non-H atoms.

**Fig. 2.**
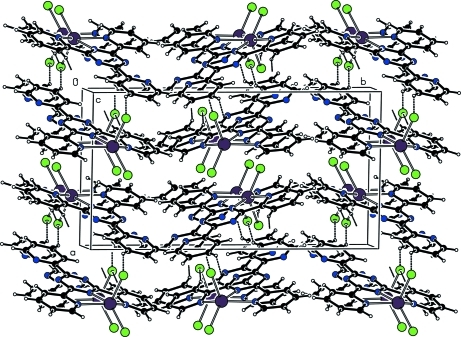
View of the unit-cell contents of the title complex. Intermolecular hydrogen-bond interactions are drawn with dashed lines.

## supplementary crystallographic information

### Comment

The crystal structure of the title complex, [MnCl_2_(tptz)] (tptz = 2,4,6-tri-2-pyridyl-1,3,5-triazine, C_18_H_12_N_6_), was previously reported in the triclinic space group *P*1 (Ha, 2010). The structure presented herein is essentially the same as the published structure and represents a monoclinic polymorph.

The Mn^II^ ion in the complex is five-coordinated in an approximately square-pyramidal geometry by three N atoms of the tridentate tptz ligand and two chloride anions (Fig. 1). While the Mn—Cl bond lengths are almost equal, the Mn—N bond lengths are somewhat different (Table 1). The Mn1—N4/6(pyridyl) bonds are slightly longer than the Mn1—N1(triazine) bond. In the crystal, the pyridyl rings are located approximately parallel to their carrier triazine ring, making dihedral angles of 5.0 (1)°, 3.8 (1)° and 3.2 (1)°. The complexes are stacked in columns along the *c* axis and linked by intermolecular C—H···Cl hydrogen bonds, forming one-dimensional chains (Fig. 2 and Table 2). There are also intramolecular C—H···N hydrogen bonds (Table 2). In the column, intermolecular π-π interactions between the six-membered rings are present, the shortest centroid-centroid distance being 3.623 (2) Å.

### Experimental

To a solution of MnCl_2_.4H_2_O (0.2969 g, 1.50 mmol) in MeOH (30 ml) was added 2,4,6-tri-2-pyridyl-1,3,5-triazine (0.1561 g, 0.50 mmol) and stirred for 3 h at room temperature. The formed precipitate was separated by filtration and washed with MeOH and dried at 50 °C, to give a yellow powder (0.1355 g). Crystals suitable for X-ray analysis were obtained by slow evaporation from an *N,N*-dimethylformamide (DMF) solution at 60 °C.

### Refinement

All H atoms were located from Fourier difference maps and refined isotropically: C—H = 0.91 (3)–0.99 (3) Å.

### Crystal data

**Table:**

[MnCl_2_(C_18_H_12_N_6_)]	*F*(000) = 884
*M**_r_* = 438.18	*D*_x_ = 1.613 Mg m^−^^3^
Monoclinic, *P*2_1_/*c*	Mo *K*α radiation, λ = 0.71073 Å
Hall symbol: -P 2ybc	Cell parameters from 3437 reflections
*a* = 10.8799 (9) Å	θ = 2.6–28.0°
*b* = 19.5524 (16) Å	µ = 1.04 mm^−^^1^
*c* = 8.6446 (7) Å	*T* = 200 K
β = 101.051 (2)°	Stick, yellow
*V* = 1804.8 (3) Å^3^	0.29 × 0.15 × 0.15 mm
*Z* = 4	

### Data collection

**Table:**

Bruker SMART 1000 CCD diffractometer	4450 independent reflections
Radiation source: fine-focus sealed tube	2653 reflections with *I* > 2σ(*I*)
graphite	*R*_int_ = 0.056
φ and ω scans	θ_max_ = 28.3°, θ_min_ = 1.9°
Absorption correction: multi-scan (*SADABS*; Bruker, 2000)	*h* = −14→13
*T*_min_ = 0.826, *T*_max_ = 1.000	*k* = −26→21
13299 measured reflections	*l* = −11→11

### Refinement

**Table:**

Refinement on *F*^2^	Primary atom site location: structure-invariant direct methods
Least-squares matrix: full	Secondary atom site location: difference Fourier map
*R*[*F*^2^ > 2σ(*F*^2^)] = 0.045	Hydrogen site location: inferred from neighbouring sites
*wR*(*F*^2^) = 0.116	All H-atom parameters refined
*S* = 1.01	*w* = 1/[σ^2^(*F*_o_^2^) + (0.0443*P*)^2^] where *P* = (*F*_o_^2^ + 2*F*_c_^2^)/3
4450 reflections	(Δ/σ)_max_ < 0.001
292 parameters	Δρ_max_ = 0.52 e Å^−^^3^
0 restraints	Δρ_min_ = −0.56 e Å^−^^3^

### Special details

**Table:**

Geometry. All e.s.d.'s (except the e.s.d. in the dihedral angle between two l.s. planes) are estimated using the full covariance matrix. The cell e.s.d.'s are taken into account individually in the estimation of e.s.d.'s in distances, angles and torsion angles; correlations between e.s.d.'s in cell parameters are only used when they are defined by crystal symmetry. An approximate (isotropic) treatment of cell e.s.d.'s is used for estimating e.s.d.'s involving l.s. planes.
Refinement. Refinement of *F*^2^ against ALL reflections. The weighted *R*-factor *wR* and goodness of fit *S* are based on *F*^2^, conventional *R*-factors *R* are based on *F*, with *F* set to zero for negative *F*^2^. The threshold expression of *F*^2^ > σ(*F*^2^) is used only for calculating *R*-factors(gt) *etc*. and is not relevant to the choice of reflections for refinement. *R*-factors based on *F*^2^ are statistically about twice as large as those based on *F*, and *R*- factors based on ALL data will be even larger.

### Fractional atomic coordinates and isotropic or equivalent isotropic displacement parameters (Å^2^)

**Table:**

	*x*	*y*	*z*	*U*_iso_*/*U*_eq_	
Mn1	0.67220 (5)	−0.04692 (2)	0.39757 (5)	0.02899 (15)	
Cl1	0.85416 (9)	−0.08835 (5)	0.32140 (10)	0.0471 (3)	
Cl2	0.49434 (8)	−0.09448 (4)	0.23876 (10)	0.0428 (2)	
N1	0.7359 (2)	0.01546 (12)	0.6079 (3)	0.0259 (6)	
N2	0.8167 (2)	0.12055 (12)	0.7203 (3)	0.0266 (6)	
N3	0.8117 (2)	0.02159 (12)	0.8809 (3)	0.0279 (6)	
N4	0.6701 (2)	0.06745 (12)	0.3264 (3)	0.0279 (6)	
N5	0.9360 (2)	0.18914 (13)	0.9898 (3)	0.0324 (6)	
N6	0.6586 (2)	−0.11029 (12)	0.6245 (3)	0.0277 (6)	
C1	0.7629 (3)	0.08207 (14)	0.5988 (3)	0.0256 (7)	
C2	0.7235 (3)	0.11228 (15)	0.4397 (3)	0.0255 (7)	
C3	0.7402 (3)	0.18052 (16)	0.4105 (4)	0.0286 (7)	
H3	0.783 (3)	0.2070 (16)	0.499 (4)	0.046 (10)*	
C4	0.7004 (3)	0.20518 (17)	0.2583 (4)	0.0340 (8)	
H4	0.710 (3)	0.2521 (15)	0.233 (4)	0.034 (9)*	
C5	0.6451 (3)	0.16038 (17)	0.1432 (4)	0.0342 (8)	
H5	0.614 (3)	0.1737 (14)	0.039 (4)	0.029 (8)*	
C6	0.6321 (3)	0.09226 (17)	0.1815 (4)	0.0326 (8)	
H6	0.595 (3)	0.0583 (15)	0.109 (4)	0.036 (9)*	
C7	0.8439 (3)	0.08676 (14)	0.8588 (3)	0.0249 (7)	
C8	0.9099 (3)	0.12252 (15)	1.0019 (3)	0.0259 (7)	
C9	0.9396 (3)	0.08632 (17)	1.1435 (4)	0.0304 (7)	
H9	0.922 (3)	0.0405 (14)	1.147 (3)	0.027 (8)*	
C10	0.9995 (3)	0.12008 (19)	1.2765 (4)	0.0352 (8)	
H10	1.016 (3)	0.0986 (15)	1.372 (4)	0.041 (10)*	
C11	1.0281 (3)	0.18794 (18)	1.2663 (4)	0.0354 (8)	
H11	1.067 (3)	0.2127 (17)	1.357 (4)	0.057 (11)*	
C12	0.9943 (3)	0.22022 (18)	1.1217 (4)	0.0358 (8)	
H12	1.007 (3)	0.2657 (15)	1.110 (3)	0.026 (8)*	
C13	0.7578 (3)	−0.01119 (14)	0.7514 (4)	0.0268 (7)	
C14	0.7155 (3)	−0.08276 (14)	0.7630 (3)	0.0261 (7)	
C15	0.7327 (3)	−0.11732 (17)	0.9051 (4)	0.0328 (8)	
H15	0.779 (3)	−0.0970 (15)	0.993 (4)	0.042 (10)*	
C16	0.6929 (3)	−0.18411 (18)	0.9038 (4)	0.0413 (9)	
H16	0.699 (3)	−0.2114 (16)	0.992 (4)	0.046 (10)*	
C17	0.6338 (3)	−0.21370 (17)	0.7629 (4)	0.0384 (8)	
H17	0.606 (3)	−0.2621 (15)	0.760 (3)	0.035 (9)*	
C18	0.6168 (3)	−0.17449 (16)	0.6269 (4)	0.0322 (7)	
H18	0.567 (3)	−0.1904 (15)	0.528 (4)	0.033 (9)*	

### Atomic displacement parameters (Å^2^)

**Table:**

	*U*^11^	*U*^22^	*U*^33^	*U*^12^	*U*^13^	*U*^23^
Mn1	0.0372 (3)	0.0270 (3)	0.0217 (3)	0.0009 (2)	0.0029 (2)	−0.0030 (2)
Cl1	0.0452 (6)	0.0684 (7)	0.0269 (5)	0.0200 (4)	0.0046 (4)	0.0010 (4)
Cl2	0.0392 (5)	0.0497 (6)	0.0354 (5)	−0.0031 (4)	−0.0029 (4)	−0.0097 (4)
N1	0.0348 (15)	0.0224 (13)	0.0199 (13)	0.0009 (11)	0.0035 (11)	−0.0001 (10)
N2	0.0296 (15)	0.0277 (14)	0.0218 (13)	0.0020 (11)	0.0028 (11)	−0.0001 (11)
N3	0.0363 (16)	0.0248 (14)	0.0213 (13)	0.0008 (11)	0.0023 (12)	−0.0017 (11)
N4	0.0333 (16)	0.0282 (14)	0.0206 (14)	−0.0012 (11)	0.0013 (11)	−0.0014 (11)
N5	0.0366 (17)	0.0307 (15)	0.0277 (15)	−0.0004 (12)	0.0006 (12)	−0.0028 (12)
N6	0.0339 (16)	0.0255 (14)	0.0228 (14)	0.0029 (11)	0.0033 (11)	−0.0021 (11)
C1	0.0284 (17)	0.0246 (17)	0.0234 (16)	−0.0004 (13)	0.0038 (13)	−0.0024 (12)
C2	0.0293 (17)	0.0269 (17)	0.0205 (15)	0.0014 (13)	0.0053 (13)	−0.0006 (12)
C3	0.0315 (18)	0.0301 (18)	0.0225 (16)	−0.0002 (14)	0.0007 (14)	−0.0017 (13)
C4	0.044 (2)	0.0272 (19)	0.0319 (19)	0.0023 (15)	0.0090 (16)	0.0064 (14)
C5	0.042 (2)	0.040 (2)	0.0195 (17)	0.0028 (15)	0.0020 (15)	0.0034 (14)
C6	0.041 (2)	0.035 (2)	0.0206 (17)	0.0009 (15)	0.0028 (15)	−0.0031 (14)
C7	0.0252 (17)	0.0241 (17)	0.0244 (16)	0.0034 (12)	0.0020 (13)	−0.0003 (12)
C8	0.0254 (17)	0.0277 (17)	0.0231 (16)	0.0052 (13)	0.0005 (13)	−0.0004 (13)
C9	0.0343 (19)	0.0307 (19)	0.0262 (18)	0.0032 (14)	0.0054 (15)	0.0015 (14)
C10	0.036 (2)	0.048 (2)	0.0194 (18)	0.0072 (16)	0.0010 (15)	0.0007 (15)
C11	0.032 (2)	0.047 (2)	0.0252 (18)	0.0034 (15)	0.0009 (15)	−0.0096 (16)
C12	0.037 (2)	0.031 (2)	0.038 (2)	−0.0018 (15)	0.0029 (16)	−0.0084 (16)
C13	0.0296 (17)	0.0247 (16)	0.0266 (17)	0.0030 (13)	0.0064 (14)	−0.0035 (13)
C14	0.0354 (19)	0.0230 (16)	0.0206 (16)	0.0019 (13)	0.0070 (14)	0.0004 (12)
C15	0.040 (2)	0.0314 (19)	0.0258 (18)	−0.0030 (15)	0.0039 (16)	−0.0002 (14)
C16	0.057 (3)	0.035 (2)	0.035 (2)	−0.0046 (17)	0.0146 (18)	0.0102 (17)
C17	0.052 (2)	0.0285 (19)	0.036 (2)	−0.0064 (16)	0.0116 (18)	0.0007 (15)
C18	0.036 (2)	0.0303 (19)	0.0302 (19)	−0.0014 (14)	0.0053 (15)	−0.0069 (14)

### Geometric parameters (Å, °)

**Table:**

Mn1—N1	2.189 (2)	C4—H4	0.95 (3)
Mn1—N4	2.318 (2)	C5—C6	1.386 (4)
Mn1—N6	2.348 (2)	C5—H5	0.93 (3)
Mn1—Cl1	2.3470 (10)	C6—H6	0.95 (3)
Mn1—Cl2	2.3400 (10)	C7—C8	1.481 (4)
N1—C13	1.324 (4)	C8—C9	1.397 (4)
N1—C1	1.341 (3)	C9—C10	1.377 (4)
N2—C1	1.333 (4)	C9—H9	0.92 (3)
N2—C7	1.349 (4)	C10—C11	1.369 (5)
N3—C13	1.325 (4)	C10—H10	0.91 (3)
N3—C7	1.345 (4)	C11—C12	1.386 (5)
N4—C6	1.333 (4)	C11—H11	0.95 (4)
N4—C2	1.359 (4)	C12—H12	0.91 (3)
N5—C12	1.340 (4)	C13—C14	1.483 (4)
N5—C8	1.341 (4)	C14—C15	1.383 (4)
N6—C18	1.337 (4)	C15—C16	1.375 (4)
N6—C14	1.350 (4)	C15—H15	0.92 (3)
C1—C2	1.483 (4)	C16—C17	1.390 (5)
C2—C3	1.376 (4)	C16—H16	0.92 (3)
C3—C4	1.390 (4)	C17—C18	1.386 (4)
C3—H3	0.97 (3)	C17—H17	0.99 (3)
C4—C5	1.375 (4)	C18—H18	0.97 (3)
			
N1—Mn1—N4	70.63 (9)	N4—C6—H6	112.6 (19)
N1—Mn1—Cl2	143.46 (7)	C5—C6—H6	124.3 (19)
N4—Mn1—Cl2	105.24 (7)	N3—C7—N2	124.8 (3)
N1—Mn1—Cl1	105.99 (7)	N3—C7—C8	115.2 (3)
N4—Mn1—Cl1	102.96 (7)	N2—C7—C8	119.9 (3)
Cl2—Mn1—Cl1	110.21 (4)	N5—C8—C9	122.9 (3)
N1—Mn1—N6	70.16 (8)	N5—C8—C7	118.0 (3)
N4—Mn1—N6	137.01 (8)	C9—C8—C7	119.1 (3)
Cl2—Mn1—N6	95.92 (7)	C10—C9—C8	118.8 (3)
Cl1—Mn1—N6	104.10 (6)	C10—C9—H9	120.6 (19)
C13—N1—C1	115.8 (2)	C8—C9—H9	120.5 (19)
C13—N1—Mn1	122.03 (19)	C11—C10—C9	119.2 (3)
C1—N1—Mn1	122.03 (18)	C11—C10—H10	120 (2)
C1—N2—C7	114.3 (2)	C9—C10—H10	121 (2)
C13—N3—C7	115.1 (2)	C10—C11—C12	118.4 (3)
C6—N4—C2	117.1 (3)	C10—C11—H11	121 (2)
C6—N4—Mn1	125.9 (2)	C12—C11—H11	121 (2)
C2—N4—Mn1	116.75 (19)	N5—C12—C11	124.2 (3)
C12—N5—C8	116.5 (3)	N5—C12—H12	114.1 (19)
C18—N6—C14	117.4 (3)	C11—C12—H12	121.6 (19)
C18—N6—Mn1	125.8 (2)	N1—C13—N3	124.9 (3)
C14—N6—Mn1	116.04 (18)	N1—C13—C14	115.6 (3)
N2—C1—N1	124.7 (3)	N3—C13—C14	119.5 (3)
N2—C1—C2	120.8 (3)	N6—C14—C15	123.6 (3)
N1—C1—C2	114.5 (3)	N6—C14—C13	114.3 (2)
N4—C2—C3	123.1 (3)	C15—C14—C13	122.0 (3)
N4—C2—C1	114.8 (2)	C16—C15—C14	117.9 (3)
C3—C2—C1	122.1 (3)	C16—C15—H15	123 (2)
C2—C3—C4	118.8 (3)	C14—C15—H15	119 (2)
C2—C3—H3	115.7 (19)	C15—C16—C17	119.6 (3)
C4—C3—H3	125.4 (19)	C15—C16—H16	125 (2)
C5—C4—C3	118.5 (3)	C17—C16—H16	115 (2)
C5—C4—H4	119.9 (19)	C18—C17—C16	118.5 (3)
C3—C4—H4	121.6 (19)	C18—C17—H17	120.9 (18)
C4—C5—C6	119.4 (3)	C16—C17—H17	120.6 (18)
C4—C5—H5	123.1 (18)	N6—C18—C17	122.8 (3)
C6—C5—H5	117.6 (18)	N6—C18—H18	114.8 (17)
N4—C6—C5	123.1 (3)	C17—C18—H18	122.2 (17)
			
N4—Mn1—N1—C13	173.6 (2)	C3—C4—C5—C6	−0.7 (5)
Cl2—Mn1—N1—C13	84.0 (3)	C2—N4—C6—C5	0.2 (5)
Cl1—Mn1—N1—C13	−88.0 (2)	Mn1—N4—C6—C5	−173.1 (2)
N6—Mn1—N1—C13	11.6 (2)	C4—C5—C6—N4	0.4 (5)
N4—Mn1—N1—C1	−10.8 (2)	C13—N3—C7—N2	5.2 (4)
Cl2—Mn1—N1—C1	−100.4 (2)	C13—N3—C7—C8	−177.1 (3)
Cl1—Mn1—N1—C1	87.7 (2)	C1—N2—C7—N3	−5.6 (4)
N6—Mn1—N1—C1	−172.8 (2)	C1—N2—C7—C8	176.8 (3)
N1—Mn1—N4—C6	−177.8 (3)	C12—N5—C8—C9	0.7 (4)
Cl2—Mn1—N4—C6	−35.9 (3)	C12—N5—C8—C7	179.2 (3)
Cl1—Mn1—N4—C6	79.6 (3)	N3—C7—C8—N5	−175.0 (3)
N6—Mn1—N4—C6	−152.6 (2)	N2—C7—C8—N5	2.8 (4)
N1—Mn1—N4—C2	8.9 (2)	N3—C7—C8—C9	3.5 (4)
Cl2—Mn1—N4—C2	150.78 (19)	N2—C7—C8—C9	−178.7 (3)
Cl1—Mn1—N4—C2	−93.8 (2)	N5—C8—C9—C10	−0.8 (5)
N6—Mn1—N4—C2	34.1 (3)	C7—C8—C9—C10	−179.2 (3)
N1—Mn1—N6—C18	179.1 (3)	C8—C9—C10—C11	0.1 (5)
N4—Mn1—N6—C18	153.8 (2)	C9—C10—C11—C12	0.5 (5)
Cl2—Mn1—N6—C18	33.9 (2)	C8—N5—C12—C11	−0.1 (5)
Cl1—Mn1—N6—C18	−78.7 (3)	C10—C11—C12—N5	−0.6 (5)
N1—Mn1—N6—C14	−11.4 (2)	C1—N1—C13—N3	−5.2 (4)
N4—Mn1—N6—C14	−36.7 (3)	Mn1—N1—C13—N3	170.7 (2)
Cl2—Mn1—N6—C14	−156.7 (2)	C1—N1—C13—C14	173.8 (2)
Cl1—Mn1—N6—C14	90.8 (2)	Mn1—N1—C13—C14	−10.3 (3)
C7—N2—C1—N1	0.3 (4)	C7—N3—C13—N1	0.6 (4)
C7—N2—C1—C2	178.0 (3)	C7—N3—C13—C14	−178.4 (2)
C13—N1—C1—N2	4.7 (4)	C18—N6—C14—C15	0.2 (4)
Mn1—N1—C1—N2	−171.1 (2)	Mn1—N6—C14—C15	−170.2 (2)
C13—N1—C1—C2	−173.1 (3)	C18—N6—C14—C13	−179.2 (3)
Mn1—N1—C1—C2	11.0 (3)	Mn1—N6—C14—C13	10.4 (3)
C6—N4—C2—C3	−0.6 (4)	N1—C13—C14—N6	−0.9 (4)
Mn1—N4—C2—C3	173.4 (2)	N3—C13—C14—N6	178.1 (3)
C6—N4—C2—C1	179.4 (3)	N1—C13—C14—C15	179.7 (3)
Mn1—N4—C2—C1	−6.6 (3)	N3—C13—C14—C15	−1.3 (4)
N2—C1—C2—N4	179.9 (3)	N6—C14—C15—C16	2.1 (5)
N1—C1—C2—N4	−2.2 (4)	C13—C14—C15—C16	−178.6 (3)
N2—C1—C2—C3	−0.1 (4)	C14—C15—C16—C17	−2.1 (5)
N1—C1—C2—C3	177.8 (3)	C15—C16—C17—C18	0.0 (5)
N4—C2—C3—C4	0.3 (5)	C14—N6—C18—C17	−2.5 (5)
C1—C2—C3—C4	−179.7 (3)	Mn1—N6—C18—C17	166.8 (2)
C2—C3—C4—C5	0.4 (5)	C16—C17—C18—N6	2.4 (5)

### Hydrogen-bond geometry (Å, °)

**Table:**

*D*—H···*A*	*D*—H	H···*A*	*D*···*A*	*D*—H···*A*
C10—H10···Cl1^i^	0.91 (3)	2.76 (3)	3.589 (3)	151 (3)
C15—H15···Cl1^ii^	0.92 (3)	2.81 (3)	3.633 (4)	151 (2)
C3—H3···N2	0.97 (3)	2.52 (3)	2.896 (4)	103 (2)
C9—H9···N3	0.92 (3)	2.41 (3)	2.735 (4)	101 (2)
C15—H15···N3	0.92 (3)	2.57 (3)	2.869 (4)	100 (2)

Symmetry codes: (i) −*x*+2, −*y*, −*z*+2; (ii) *x*, *y*, *z*+1.
